# ‘Forget me (not)?’ – Remembering Forget-Items Versus Un-Cued Items in Directed Forgetting

**DOI:** 10.3389/fpsyg.2015.01741

**Published:** 2015-11-16

**Authors:** Bastian Zwissler, Sebastian Schindler, Helena Fischer, Christian Plewnia, Johanna M. Kissler

**Affiliations:** ^1^Department of Psychiatry and Psychotherapy, Neurophysiology and Interventional Neuropsychiatry, University Hospital Tübingen – University of TübingenTübingen, Germany; ^2^Department of Psychology, University of BielefeldBielefeld, Germany; ^3^Center of Excellence Cognitive Interaction Technology, University of BielefeldBielefeld, Germany; ^4^Department of Psychology, University of KonstanzKonstanz, Germany

**Keywords:** episodic memory, item method, selective rehearsal, ironic process, inhibition (psychology), directed forgetting

## Abstract

Humans need to be able to selectively control their memories. This capability is often investigated in directed forgetting (DF) paradigms. In item-method DF, individual items are presented and each is followed by either a forget- or remember-instruction. On a surprise test of all items, memory is then worse for to-be-forgotten items (TBF) compared to to-be-remembered items (TBR). This is thought to result mainly from selective rehearsal of TBR, although inhibitory mechanisms also appear to be recruited by this paradigm. Here, we investigate whether the mnemonic consequences of a forget instruction differ from the ones of incidental encoding, where items are presented without a specific memory instruction. Four experiments were conducted where un-cued items (UI) were interspersed and recognition performance was compared between TBR, TBF, and UI stimuli. Accuracy was encouraged via a performance-dependent monetary bonus. Experiments varied the number of items and their presentation speed and used either letter-cues or symbolic cues. Across all experiments, including perceptually fully counterbalanced variants, memory accuracy for TBF was reduced compared to TBR, but better than for UI. Moreover, participants made consistently fewer false alarms and used a very conservative response criterion when responding to TBF stimuli. Thus, the F-cue results in active processing and reduces false alarm rate, but this does not impair recognition memory beyond an un-cued baseline condition, where only incidental encoding occurs. Theoretical implications of these findings are discussed.

## Introduction

Humans need to manage their cognitive resources in order to control their behavior. We are therefore able to ignore irrelevant stimuli and withhold pre-potent automatic responses to remain focused on a current task, although this is effortful and there are clear limits to human capacities for cognitive control (e.g., Botvinick et al., 2001). In episodic memory, as in other cognitive domains, there is constant need for selection to keep memory up-to-date with current demands. Both everyday-life and scientific research demonstrate our ability to selectively encode and retrieve memory contents (Levy and Anderson, 2009). In school, as well as in legal or more mundane contexts, we might be presented with information that we are supposed to remember as important for the future. Still, every now and then this information might turn out to be unimportant, irrelevant, or even false after presentation and we may be then told to forget it. Scientifically, variants of the directed forgetting (DF) task provide a means to study selection and updating processes in memory (Golding and MacLeod, 1998). In list-method DF, participants are shown pairs of lists and after the first list of such a pair they are instructed to either remember all items on the previous list for future testing or to forget this list. Then, in both cases a second list is presented for further learning. At the end of the experiment, unexpectedly for the participant, items from both lists are tested. The between-list forget instruction typically results in poorer memory for list 1 items and better memory for list 2 items, whereas the reverse is true following the remember instruction. Because this pattern is only apparent in free recall, but not in recognition testing, retrieval inhibition has been a dominant account for the list-method DF effect (for review, see Anderson and Hanslmayr, 2014).

In item-method DF, individual items are immediately followed by an instruction. To-be-remembered items (TBR) are followed by a ‘remember’ (R) cue while to-be-forgotten items (TBF) are followed by a ‘forget’ (F) cue. Later, memory is tested for all items, regardless of their initial instruction. This typically leads to a DF effect, better memory for TBR than for TBF. The effect is apparent both in recall and recognition (Basden and Basden, 1996) and has been shown for a variety of materials (Lehman et al., 2001; Hourihan and Taylor, 2006; Hauswald and Kissler, 2008; Hourihan et al., 2009; Quinlan et al., 2010; Nowicka et al., 2011; Zwissler et al., 2011).

Although originally thought to reflect repression in a Freudian sense (Weiner, 1968), item-method DF has been subsequently mainly attributed to selective rehearsal (Basden and Basden, 1998), assuming that TBR are rehearsed more than TBF: upon presentation, each item is held in a standby-like mode and its processing is postponed until the instruction appears. An R instruction then leads to further rehearsal, while an F instruction is supposed to terminate any further processing, leading to passive decay of the item’s representation. As a consequence, only TBR are selectively encoded and therefore better remembered than TBF.

Recent evidence suggests that participants either consciously of unconsciously make use of quite elaborate strategies to facilitate forgetting. For instance, item-method DF has been shown to interact with the loudness illusion in memory (Foster and Sahakyan, 2012): This illusion refers to the observation that when items that vary in loudness are presented for learning, participants have the subjective impression of remembering loud items better than quiet one, although objectively this is not the case (Rhodes and Castel, 2009). However, specifically in a situation where loud and quiet items are embedded in an item-method DF task, loud items are really recalled better than quiet ones. The same is not true for various control conditions, including ones that differently emphasize, via value assignment, the importance of remembering loud items, suggesting a specificity of the effect to a situation that requires intentional forgetting. Selectively rehearsing loud items, given an adequate opportunity, may be used as either an explicit or an implicit strategy to forget.

Somewhat reminiscent of the original repression account, recent behavioral evidence also demonstrates that active inhibitory processing is triggered by the forget cue in this paradigm (e.g., Fawcett and Taylor, 2010; Lee et al., 2013). Zacks and Hasher (1994) first proposed mechanisms of attentional inhibition to operate in item-method DF and a wealth of behavioral data now indicates that the instruction to forget in item-method DF amplifies effects of inhibition of return (IOR; Taylor, 2005; Taylor and Fawcett, 2011, 2012; Thompson and Taylor, 2015). Although originally thought to affect only motoric IOR magnitude (Taylor, 2005; Taylor and Fawcett, 2011, 2012), greater slowing of return to target location following F-cue than following R-cue has recently been demonstrated in both motoric and visual IOR (Thompson and Taylor, 2015). The greater IOR effect following the F-cue has been also shown to be due to genuine IOR magnification, rather than due to facilitation of reorientation to the other side (Taylor and Fawcett, 2012). Together, these data are consistent with the interpretation that inhibition of spatial attention is increased by the forget instruction. This has led to the speculation that TBF-item’s memory representations along a spatial saliency map are rendered less accessible than those of the TBR items (Thompson and Taylor, 2015). However, interactions between DF patterns and attention mechanisms seem to be paradigm-specific: whereas there is evidence that attention withdraws from forget items and reduces the processing of other information that is presented in temporal or spatial proximity (Fawcett and Taylor, 2008; Taylor and Fawcett, 2012; Lee and Hsu, 2013), very recent data demonstrates that distractibility is not generally increased following a forget instruction. For instance, reaction times to interspersed attentional orienting probes are not affected by a preceding F-cue (Taylor and Hamm, 2015).

Therefore, inhibitory mechanisms seem to be invoked by the forget instruction, but effects are paradigm-specific rather than domain-general.

Neuroscientific studies indicate more frontal and less parietal activation in response to the F- than to the R-instruction (Paz-Caballero et al., 2004; Wylie et al., 2008; van Hooff and Ford, 2011; Rizio and Dennis, 2013) as well as a positive correlation between frontal brain activity and magnitude of the DF-effect (Hauswald et al., 2011) indirectly supporting the view that some form of active inhibition is at work in item-method DF.

Whereas inhibition of spatial attention has been convincingly demonstrated in item-method DF, the mnemonic consequences have been less clearly specified. For instance, in the clinical literature the Freudian suppression metaphor is still discussed (e.g., Cottencin et al., 2006). It is clear that TBF is associated with poorer memory than TBR and that the F-instruction induces active, in the case of spatial attention also inhibitory, processing. Still, the relationship between IOR reaction time and the memory DF effect is uncertain. Fawcett and Taylor (2010) found that for successfully forgotten TBF, IOR was bigger than it was for remembered TBF, suggesting a link between the processes involved. However, this association is not reported in Taylor and Fawcett (2011) or Thompson et al. (2014).

Thus, extant evidence demonstrates that people are indeed able to selectively encode some material while ignoring, perhaps even actively inhibiting, other material presented for the same period of time. However, a different line of evidence indicates that for instance thought suppression is often ineffective and can result in paradoxical effects (Wegner et al., 1987). Regarding DF, it has been shown that prolonging cue presentation results in better memory for TBF and TBR items alike (Lee et al., 2007; Bancroft et al., 2013). This contradicts the assumption that TBF items decay passively and is also difficult to reconcile with the idea of effective memory inhibition. As a consequence the question arises, how TBF and TBR compare to a condition where items are encoded only incidentally because they are not followed by a specific memory instruction. If prolonging cue presentation improves rather than impairs memory of TBF items, suggesting that active, but not inhibitory processing is induced by TBF, how will no cue at all or an unspecific cue compare? Evidence from the Think-No Think paradigm underscores the possibility of successful intentional memory suppression of paired-associates, even below a baseline level (Anderson and Green, 2001; Anderson et al., 2004). Similarly, automatic memory inhibition of some items below a given baseline has been shown for the retrieval-induced forgetting paradigm (Anderson et al., 1994).

A wealth of research on thought control mechanisms has demonstrated ironic processes when people try to suppress their thoughts (Wegner et al., 1987; Wegner, 1994, 1997; Wenzlaff and Wegner, 2000), although there are important differences between thought suppression and item-method DF paradigms. For instance, in ironic thought control the effect disappears when alternative thoughts are instructed. Still, by analogy, in item-method DF, any cue might initially re-orient participants to the preceding stimulus. If TBF cues were perceived as ‘suppress’ commands, the success and behavioral consequences of such suppression attempts might be uncertain (Wegner et al., 1987; Wegner, 1994, 1997; Wenzlaff and Wegner, 2000), although the presence of other items to which processing resources could be redirected may counteract any ironic processes.

Here, we addressed the status of forget items in item method DF by introducing un-cued items (UI) into the paradigm. We tested, whether memory for TBF is equally bad (selective rehearsal) or perhaps even worse (memory inhibition) than if no instruction were given, and items were only incidentally encoded. The presence of UI may provide participants to redirect their processing resources to these items, further reducing TBF encoding. If, however, F-cues initiate re-alerting (or ironic monitoring as found in thought suppression research), TBF could still be actively processed and highlighted to a certain extent. In that case UI would be remembered worse than both TBR and also TBF.

As in several previous studies we use a recognition memory design with complex pictorial stimuli and similar paired distracters (Quinlan et al., 2010; Hauswald et al., 2011; Nowicka et al., 2011; Zwissler et al., 2011). This facilitates a separate analysis of recognition accuracy and response bias. We have been using picture stimuli in an effort to obtain more language- and culture-independent results and in order to be able to work with linguistically heterogeneous clinical populations (e.g., Zwissler et al., 2012; Baumann et al., 2013). So far, the basic mechanism of selective rehearsal has been shown to apply also to pictorial stimuli (Hourihan et al., 2009), but differences may exist precluding generalization of results to studies using word stimuli.

To increase motivation to show full effort on the final test, participants received a performance-dependent monetary bonus encouraging performance accuracy (see also MacLeod, 1999).

We expect differential processing of TBR, TBF, and UI items to be reflected in memory performance. Selective rehearsal should improve recognition accuracy for TBR over both TBF and UI. We test, whether memory accuracy differs between incidental encoding of UI and intentional forgetting as instructed for TBF. The different instructions also could affect participants’ readiness to respond to an item given a similar amount of mnemonic information. This would be reflected in distinct response biases: Because strengthening an item’s memory representation leads to a more conservative response bias (Hirshman, 1995), according to selective rehearsal, TBR items should be responded to most conservatively. If TBF cues prompt a distinct, potentially inhibitory effect on response criterion setting, response bias for TBF items should be most conservative.

To investigate the effect of implicit encoding in item method DF, the fate of TBF items is compared with both TBR and UI items. Four experiments were conducted: Experiment 1 presents a basic comparison of recognition memory for the three item types, Experiment 2 uses a longer item list, and Experiment 3 replaces the instructions by three symbolic cues, addressing the possibility that physical cue characteristics affect performance. Experiment 4 tests the effects of symbolic cues with a different item set.

## Experiment 1

### Method

#### Participants

Thirty-one students at the University of Konstanz, Germany, (24 women; mean age = 21.67, *SE* = 0.44; range: 18–28 years) participated in return for course credit or 3 texteuro basic compensation. They could earn additional performance-dependent bonus. In all experiments, participants gave written informed consent and the research was conducted in accordance with the Declaration of Helsinki. The experiment was approved by the Ethics Committee of the University of Konstanz.

#### Stimuli

**FIGURE 1:**
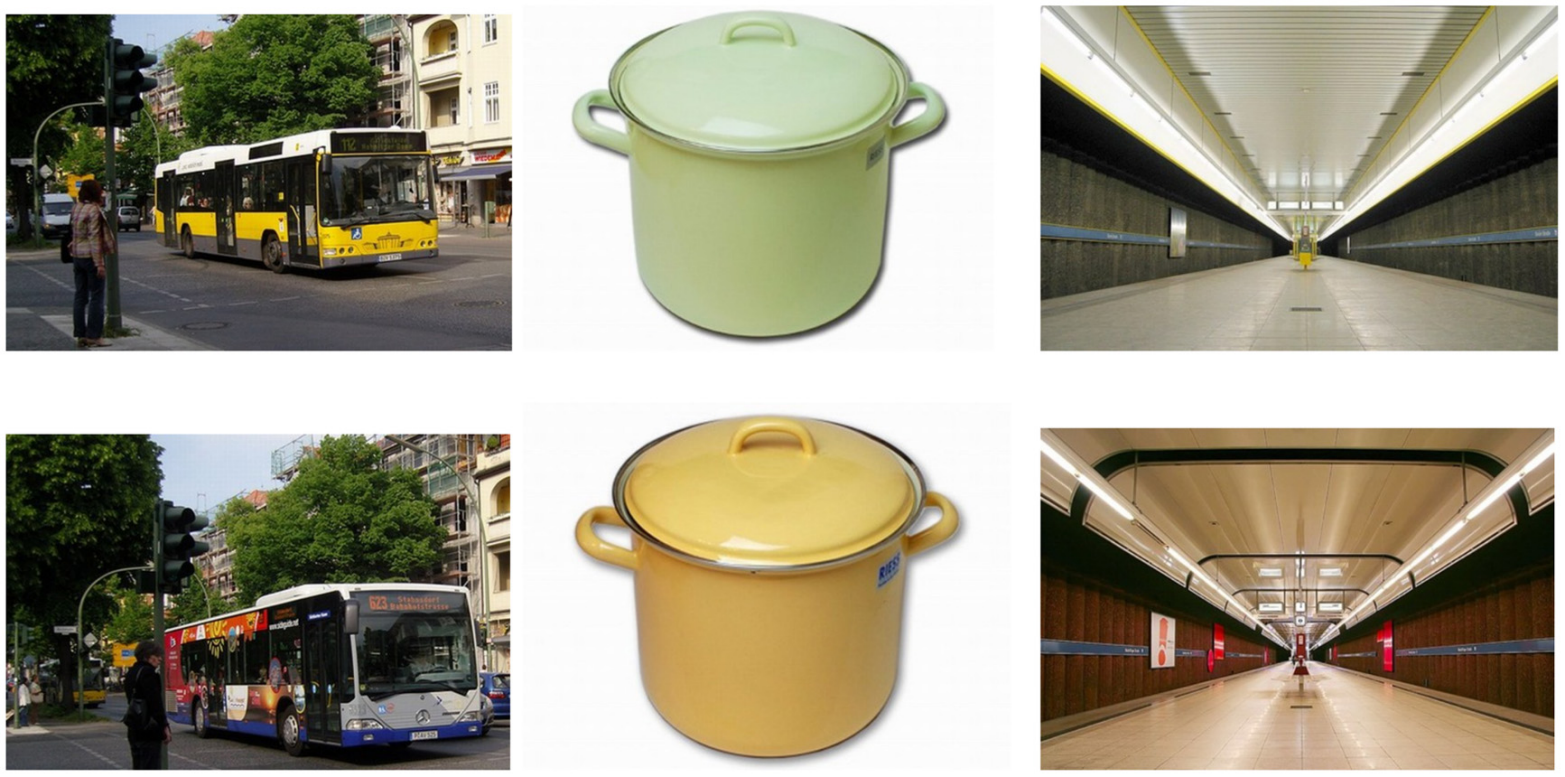
**Illustration of the picture sets for experiments 1–3 showing three representative target-distracter pairs**.

Seventy-five target-distracter pairs of images were used for memory testing. Pairs were thematically unique within the set and differed only in perceptual detail (see **Figure 1** for examples), thus allowing for a separate analysis of hits and false alarms in response to the differently cued items. The images showed people, landscapes, animals, or social scenes. One member of each pair was assigned to each of two sets (A and B), image-set assignment was counterbalanced, and image-cue assignment was randomized. During learning, all set A images were presented in random order. During recognition, all images from both sets were shown at random, set B images serving as related lures.

#### Procedure: Learning Phase

Participants were explained that they would be presented pictures some of which would be relevant to successful task performance and others would not. Relevant pictures would be followed by either a ‘remember it’ (R) cue or by a ‘forget it’ (F) cue. Irrelevant pictures were not further instructed (‘un-cued’ ∼ U). The exact wording of the instruction was: “You will see a series of pictures. Some will be followed by a ‘MMM’ cue. Then it is important to remember the preceding picture for later testing. Some will be followed by a ‘VVV’ cue. Then it is important to forget the preceding picture. Some pictures will not be followed by a cue.” Up front, there was no instruction on how to behave in response to items that were not followed by a cue. If participants asked what the purpose of the un-cued pictures was, they were told that these served to ensure stable time lags between the cued pictures in a subsequent imaging study. Then, all pictures from one set were shown in sequence, each for 2 s. Immediately after each picture either the F instruction symbolized by ‘VVV’ (‘vergessen’ ∼ ‘forget’), the R instruction signaled by ‘MMM’ (‘merken’ ∼ ‘remember’) or a blank screen appeared for 2 s. Then, a fixation cross was presented for 1 s, after which the next picture was shown.

After learning, a break of 10 min took place during which participants were asked to perform a speeded attention endurance test (d2; Brickenkamp, 1994) to ensure that they did not further rehearse the material. This paper–pencil test requires participants to identify and mark target symbols embedded among similar distracter symbols.

#### Procedure: Recognition Phase

Before the recognition test, participants were told that they now should try to accurately recognize ALL initially presented images, regardless of their previous instruction and that they could earn 0.2 yesu1 for each correctly recognized picture, but would lose the same amount for false alarms, perfect performance resulting in a maximum of 15 yesu1 (75 × 0.2 yesu1). Thereby, recognition accuracy was reinforced and guessing was discouraged.

During the test, a random sequence of the 75 old and 75 similar new pictures (thematically paired distracters), was administered. Each picture was shown for 300 ms and participants were asked to decide by button press whether they had seen it before. Presentation time for recognition was kept short to encourage spontaneous responses, but reaction time was not limited. After each response, a fixation cross was presented for 700 ms before the next picture appeared. Experimental material was presented on a laptop computer (Dell Latitude D830) using Presentation Software (Neurobehavioral Systems Inc., Albany, NY, USA). Upon completion of the experiment, participants were paid and debriefed about the purpose of the study.

#### Statistical Analyses

Statistical calculations were performed with SPSS 20.0 (SPSS Inc., www.spss.com). Data were analyzed using repeated-measures ANOVAs with the within-factor cue (Forget, Remember, Un-cued). ANOVAs were calculated for hits and false alarms, as well as for discrimination accuracy and response bias. *Post hoc* comparisons were calculated with an alpha level of 0.05 using Fisher’s Least Significant Differences test. If the sphericity assumption was violated, degrees of freedom were corrected according to Greenhouse–Geisser.

### Results

#### Hits and False Alarms

**Table 1** presents mean hit and false alarm rates in the ‘remember,’ ‘forget,’ and ‘un-cued’ conditions for the first experiment. A significant main effect was found on hits [*F*_(2,60)_ = 20.78; *p* < 0.001; $\eta_{p}^{2}$ = 0.41]. *Post hoc* comparisons showed that for hit rate was highest for TBR, being significantly higher than TBF (*p* < 0.01) and UI (*p* < 0.001). Hit rate for TBF was also higher than for UI (*p* < 0.01). Further, there was also a significant main effect for false alarms [*F*_(2,60)_ = 7.91; *p* = 0.001; $\eta_{p}^{2}$ = 0.21]. *Post hoc* comparisons showed that the false alarm rate was considerably higher for UI lures than for TBF lures (*p* < 0.01) and TBR lures (*p* < 0.01), the latter two not differing (*p* = 0.82).

**Table 1:** Experiment 1.

Hits			False Alarms		
	
Remember	Forget	Un-cued	Remember	Forget	Un-cued
0.77^a^	0.66^b^	0.61^c^	0.14^a^	0.14^a^	0.22^b^
(0.02)	(0.04)	(0.03)	(0.01)	(0.02)	(0.02)


*Mean hit and false alarm rates in the ‘remember,’ ‘forget,’ and ‘un-cued’ condition. Standard errors appear in parentheses below means; means in the same row sharing the same superscript letter do not differ significantly from one each other, means that do not share superscripts differ at *p* ≤ 0.05.*

#### Discrimination Accuracy and Response Bias

**FIGURE 2:**
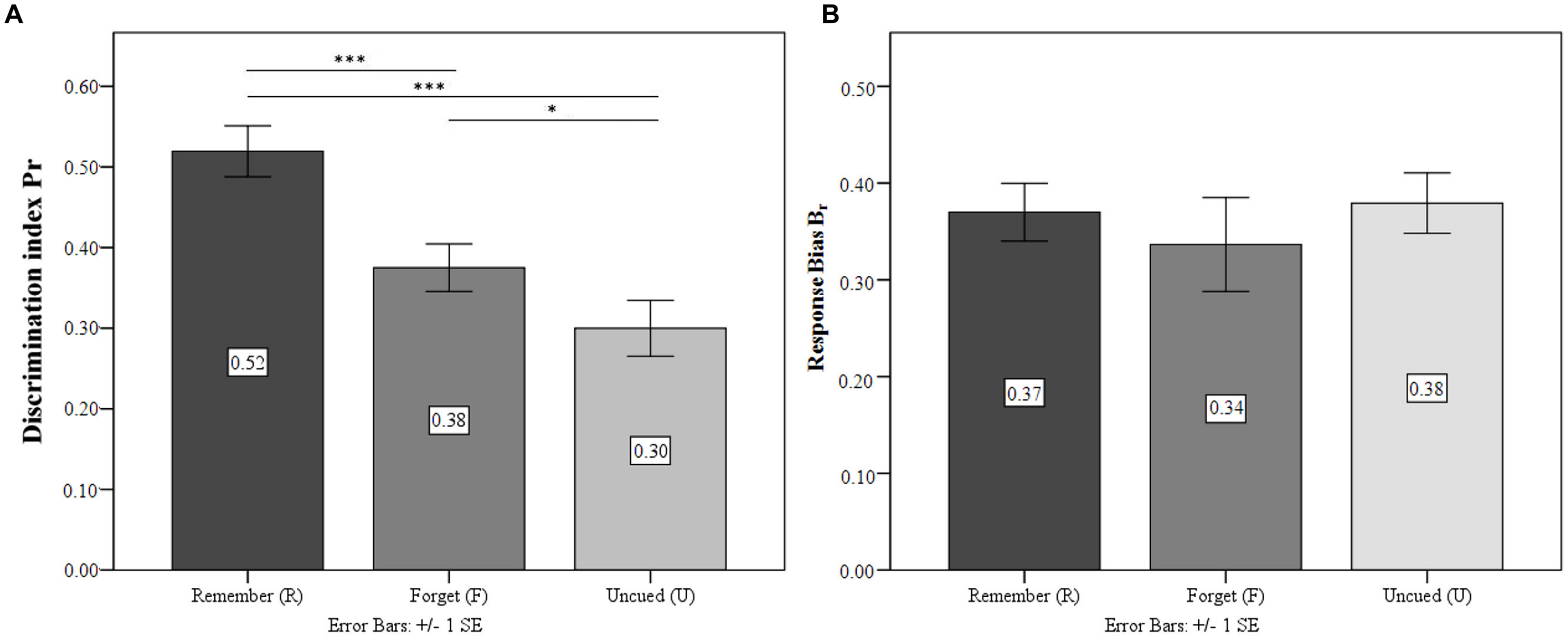
**Experiment 1.** **(A)** Discrimination index for the condition Remember (R), Forget (F), and Un-cued (U). **(B)** Response bias for the three conditions. ^∗^*p* ≤ 0.05, ^∗∗∗^*p* ≤ 0.001.

Following Snodgrass and Corwin’s (1988) two-high-threshold model, discrimination accuracy (*P_r_* = hit rate – false alarm rate) and response bias [*B*_r_ = false alarm rate/(1 – *P*_r_)] were analyzed from the data, simultaneously taking into account hits and false alarms and resulting in separate measures of recognition accuracy and response bias in DF.^1^ ANOVA confirmed significant differences in the discrimination of differently cued stimuli [*F*_(2,60)_ = 28.69; *p* < 0.001; $\eta_{p}^{2}$ = 0.49] and revealed that TBR were recognized significantly more accurately than both TBF (*p* < 0.01) and UI (*p* < 0.001). Crucially, *P*_r_ was significantly higher for TBF than for UI (*p* < 0.001). There was no significant effect on recognition bias *B*_r_ [*F*_(1.67,50.22)_ = 0.53; *p* = 0.56; $\eta_{p}^{2}$ = 0.02]. In general, response bias was rather conservative (*B*_r_ ranging from 0.34 to 0.38). Results are depicted in **Figure 2**.

### Discussion Experiment 1

Experiment 1 indicates that in item-method DF, presenting stimuli for incidental encoding with no specific instruction results in poorer memory accuracy than both a remember and a forget instruction. This is inconsistent with the notion of successful memory inhibition in item-method DF. As expected and in line with selective rehearsal, TBR were recognized more accurately than TBF or UI. Moreover, TBF were also recognized more accurately than UI, implying the possibility of ironic effects (Wegner et al., 1987; Wegner, 1994, 1997; Wenzlaff and Wegner, 2000). Results indicate that while selective rehearsal may account for TBR memory superiority, TBF seem to trigger active, non-inhibitory, memory processing that exceeds the one of completely un-cued, incidentally encoded, items. To further investigate this, a second experiment is conducted using more pictures and reducing picture presentation duration, thus increasing task difficulty. This addresses the possibility that, in spite of a monetary incentive to the contrary, participants somehow remembered list A items in association with their instruction and were guided by this on the recognition test.

## Experiment 2

### Method

The experimental methods mirrored the ones used in Experiment 1 with the following exceptions:

#### Participants

Forty-one students took part (25 women; mean age = 23.02, *SE* = 1.00; range: 18–56 years). They were recruited by posters around University of Konstanz campus. The experiment was approved by the Ethics Committee of the University of Konstanz.

#### Stimuli

The stimulus set was expanded to 90 target-distracter pairs of similar pictures.

#### Procedure: Learning Phase

Presentation duration was reduced to one second.

### Results

#### Hits and False Alarms

**Table 2** presents mean hit and false alarm rates in the ‘remember,’ ‘forget,’ and ‘un-cued’ conditions for the second experiment. A significant main effect was observed for hits [*F*_(2,80)_ = 31.57; *p* < 0.001; $\eta_{p}^{2}$ = 0.44]. *Post hoc* comparisons showed that hit rate was highest for TBR, being significantly higher than TBF (*p* < 0.001) and UI (*p* < 0.001). The latter two did not differ (*p* = 0.24). Further, there was a significant main effect for false alarms [*F*_(2,80)_ = 16.63; *p* < 0.001; $\eta_{p}^{2}$ = 0.29]. *Post hoc* comparisons showed that the false alarm rate was lowest for TBF, false alarms for TBF being significantly lower than TBR (*p* < 0.01) and UI (*p* < 0.001). False Alarm rate was also significantly lower for TBR than UI (*p* < 0.05).

**Table 2:** Experiment 2.

Hits			False Alarms		
	
Remember	Forget	Un-cued	Remember	Forget	Un-cued
0.73^a^	0.59^b^	0.57^b^	0.28^a^	0.22^b^	0.32^c^
(0.02)	(0.03)	(0.03)	(0.02)	(0.02)	(0.02)


*Mean hit and false alarm rates in the ‘remember,’ ‘forget,’ and ‘un-cued’ condition. Standard errors appear in parentheses below means; means in the same row sharing the same superscript letter do not differ significantly from each other, means that do not share superscripts differ at *p* ≤ 0.05.*

#### Discrimination Accuracy and Response Bias

**FIGURE 3:**
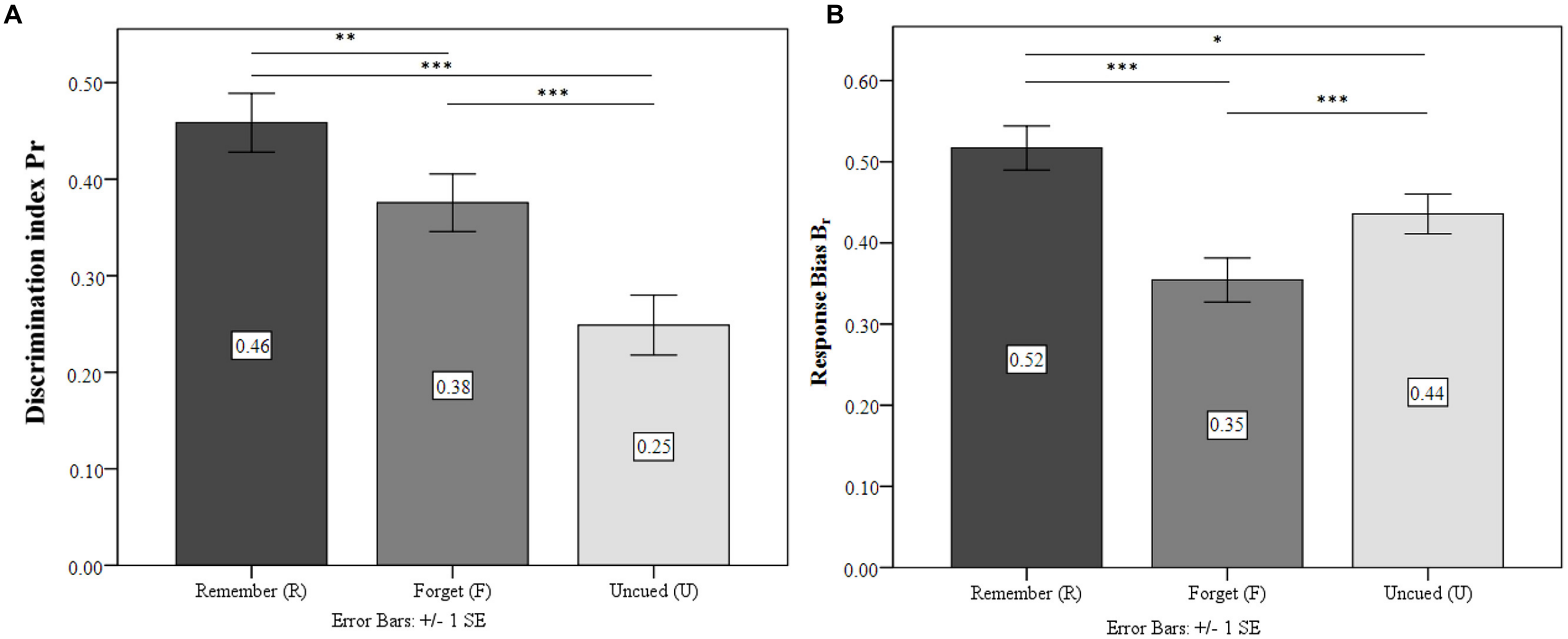
**Experiment 2.** **(A)** Discrimination index for the condition Remember (R), Forget (F), and Un-cued (U). **(B)** Response bias for the three conditions. ^∗^*p* ≤ 0.05, ^∗∗^*p* ≤ 0.01, ^∗∗∗^*p* ≤ 0.001.

Repeated measures ANOVA confirmed significant differences in the discrimination accuracy *P*_r_ of the differently cued stimuli [*F*_(1.75,69.82)_ = 33.71; *p* < 0.001; $\eta_{p}^{2}$ = 0.46]. TBR were recognized significantly more accurately than both TBF and UI (both *p* < 0.001; see **Figure 3A**). Crucially, *P*_r_ was significantly higher for TBF than for UI (*p* < 0.001). There were also significant differences for the response bias *B*_r_ [*F*_(1.41,56.47)_ = 18.05; *p* < 0.001; $\eta_{p}^{2}$ = 0.31]. TBF response bias was significantly more conservative than TBR and UI (both *p* < 0.001, see **Figure 3B**). TBR showed a more liberal response bias than UI (*p* < 0.05).

### Discussion Experiment 2

As in Experiment 1, higher recognition accuracy was found for TBR compared to TBF or UI items. Again, TBF were recognized more accurately than UI, overall confirming that selective rehearsal can account for the TBR advantage and that TBF induces, active, albeit for recognition memory seemingly non-inhibitory, processing. As a new finding, in this longer version instruction affected response bias: TBF were responded to more conservatively than TBR and UI, TBR being more liberal than UI. Also, it has to be noted that unlike in Experiment 1, the effect is now driven more by instruction-induced changes in false alarms than in hits, requiring further scrutiny. Possibly, because presentation time during learning was reduced, participants relied more on gist representation, bringing up overall false alarm rate and increasing its contribution to the effects. Interestingly, false alarm rates were across both experiments lower for TBF than for UI and in Experiment 2 also lower for TBF than for TBR. However, a possible limitation of both experiments is that in the UI condition only a blank screen was used, resulting in a perceptual difference from the other two conditions. Explicit processing cues may automatically induce reprocessing of the previously presented picture for both cued item types as participants may need to refresh the cue-item association to initiate further active processing, thus causing superior memory for perceptually cued items in comparison with items for which no cue appears and that after the initial rehearsal phase are allowed to passively decay. On the other hand, the absence of a cue may also result in UI items being on average rehearsed a little longer until participants realize that there will be no cue. If so, the latter possibility should reduce differences between R, F, and U, whereas the former should enlarge it. To further examine the pattern of results and ensure that variation in perceptual input had no impact on the current results, a third experiment was performed using symbolic cues for all three conditions.

## Experiment 3^2^

### Method

Experiment 3^2^ resembled Experiment 2 with the following exceptions:

#### Participants

Twenty-seven students (14 women; mean age = 24.23, *SE* = 0.55; range: 19–32 years) from the University of Tübingen, Germany, participated. The experiment was approved by the Ethics Committee of the University of Tübingen.

#### Procedure: Learning Phase

The letter-cues were replaced by symbolic cues. A blue circle, a purple square and yellow triangle were randomly assigned to represent R, F, or U. Symbol-cue assignment was counterbalanced across participants. The basic procedure was identical to Experiment 2.

### Results

Twenty-six data-sets were available for analysis as data from one participant were lost.

#### Hits and False Alarms

A significant main effect was observed for hits [*F*_(2,50)_ = 22.51; *p* < 0.001; $\eta_{p}^{2}$ = 0.47]. TBR hit rate was significantly higher than TBF and UI hit rates (*p* < 0.001, respectively), whereas TBF and UI did not differ (*p* = 0.80). A significant main effect for false alarms was also found [*F*_(2,50)_ = 16.23; *p* < 0.001; $\eta_{p}^{2}$ = 0.39]. *Post hoc* comparisons showed that the false alarm rate was significantly higher for UI than for TBR (*p* < 0.01) and TBF (*p* < 0.001), while TBR tended to be higher than TBF (*p* = 0.06). Mean hit and false-alarm rates are given in **Table 3**.

**Table 3:** Experiment 3.

Hits			False Alarms		
	
Remember	Forget	Un-cued	Remember	Forget	Un-cued
0.73^a^	0.57^b^	0.57^b^	0.24^a^	0.20^a^	0.32^b^
(0.02)	(0.03)	(0.03)	(0.02)	(0.02)	(0.03)


*Mean hit and false alarm rates in the ‘remember,’ ‘forget,’ and ‘un-cued’ condition. Standard errors appear in parentheses below means; means in the same row sharing the same superscript letter do not differ significantly from each other, means that do not share superscripts differ at *p* ≤ 0.05.*

#### Discrimination Accuracy and Response Bias

**FIGURE 4:**
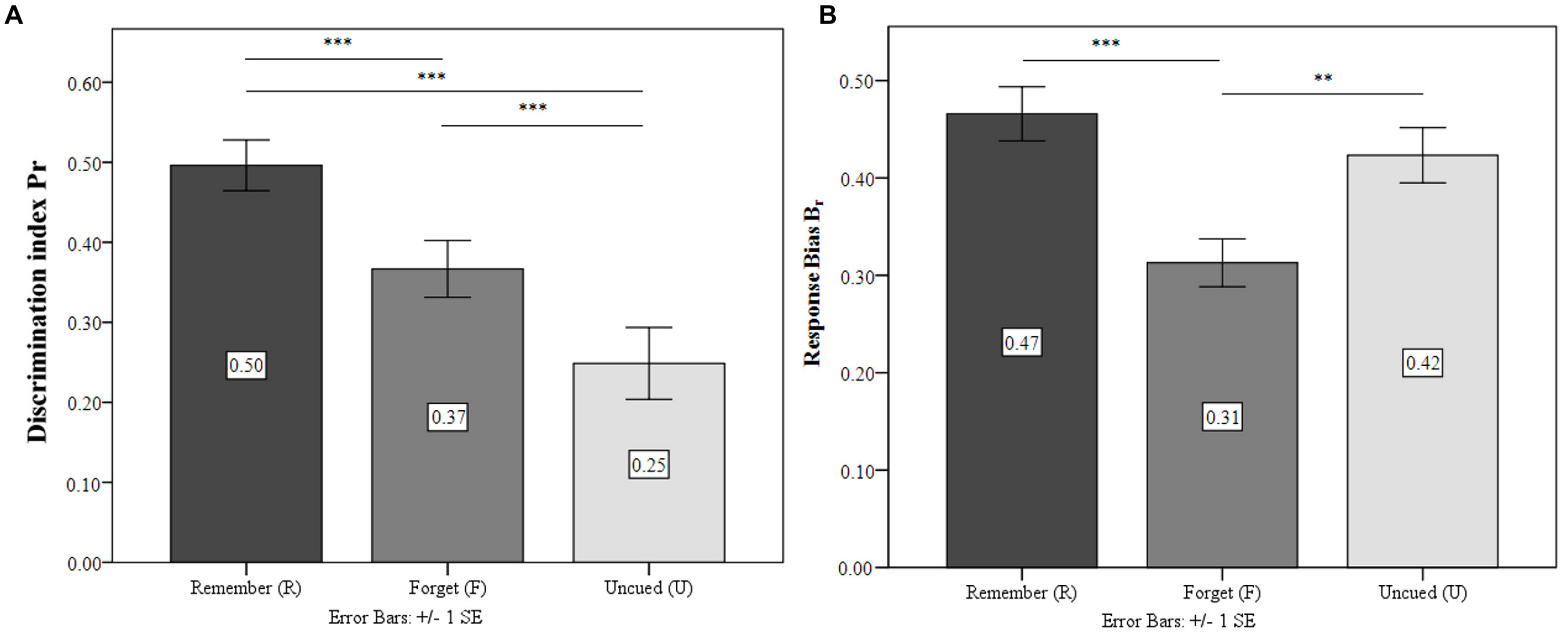
**Experiment 3.** **(A)** Discrimination index for the condition Remember (R), Forget (F), and Un-cued (U). **(B)** Response bias for the three conditions. ^∗∗^*p* ≤ 0.01, ^∗∗∗^*p* ≤ 0.001.

ANOVA confirmed significant differences in discrimination accuracy *P*_r_ between differently cued stimuli [*F*_(2,50)_ = 25.08; *p* < 0.001; $\eta_{p}^{2}$ = 0.50] and revealed that TBR were recognized significantly more accurately than TBF (*p* < 0.01) and UI (*p* < 0.001). Also, TBF recognition was higher than UI (*p* < 0.01). As in Experiment 2, there were also significant differences for recognition bias *B*_r_ [*F*_(2,50)_ = 16.50; *p* < 0.001; $\eta_{p}^{2}$ = 0.40]. TBF response bias was significantly more conservative than TBR (*p* < 0.001) and UI (*p* < 0.001). TBR and UI did not differ (*p* = 0.19). **Figure 4** illustrates this pattern.

### Discussion Experiment 3

Experiment 3 replicates findings from Experiments 1 and 2 regarding response accuracy. Furthermore, by introducing a third (symbolic) cue in addition to F and R, a potential weakness of the two previous experiments was addressed. Therefore, the pattern cannot be explained by differences in the physical features of the cues, or by the fact that F and R cues induced re-processing, whereas UI did not. It rather has to be assumed that a negative instruction leads to a more accurate representation of the respective stimulus compared to no instruction at all, although both conditions are perceptually identical. Regarding materials, Experiment 3 is directly comparable with Experiment 2, and in both the accuracy effect is carried more by false alarms than by hit rate. In both these experiments, fewest false alarms are made for TBF items and effects on recognition bias are observed with R stimuli being classified almost without bias, U stimuli slightly more conservatively and F stimuli most conservatively. This difference from Experiment 1 may result from increasing task difficulty and participants’ greater reliance on gist representation. Experiments 2 and 3 used more stimuli and a faster presentation rate, resulting in overall lower hit and higher false alarm rates. The response bias results depart from the commonly observed pattern that strengthening items leads to a more conservative response bias (e.g., Hirshman, 1995; Stretch and Wixted, 1998). The initial forget instruction may induce a subjective underrepresentation of the frequency of forget items on the test list (Strack and Förster, 1995; Hirshman and Henzler, 1998), reducing participants’ readiness to respond to these items. If so, such a subjective underrepresentation appears not to be due to variations in perceptual input between Experiments 2 and 3 as the pattern was very similar and if anything, one might expect items associated with less perceptual input (UI in Experiment 2) to be more prone to subjective underrepresentation. There might be a small perceptual effect, since in Experiment 2 the response bias for TBR is significantly higher than for UI and this difference disappears in Experiment 3. However, in terms of response bias, the comparison with TBF items is the same in both experiments. Still, in Experiments 1 and 2 forget and remember conditions differed perceptually from the un-cued condition. Although so far this perceptual variation does not seem to impact the pattern of results in a major way, a fourth experiment was conducted to replicate the symbolic cue effect. In this fourth experiment some of the previous picture pairs were replaced with new pairs because several participants had indicated that they found some of the target-distracter pairs too similar and easily confusable (see **Figure 1**). If so, this would have added additional noise to the data, assuming that these pairs had been randomly distributed across the conditions as implemented by the random picture-condition assignment. However, if distribution of these pairs had been uneven across conditions this could even have affected the pattern of results.

## Experiment 4

Experiment 4 recorded both behavioral and EEG data. EEG data will be fully reported elsewhere. Behaviourally, Experiment 4 resembled Experiment 3 with the following exceptions:

### Method

#### Participants

Twenty-four students (14 women; mean age = 22.79, *SE* = 0.93; range: 19–35 years) from the University of Bielefeld participated. Performance-dependent bonus was 5 ct per correct item, adding up to a maximum bonus of yesu1 5. The experiment was approved by the Ethics Committee of the University of Bielefeld.

#### Stimuli

**FIGURE 5:**
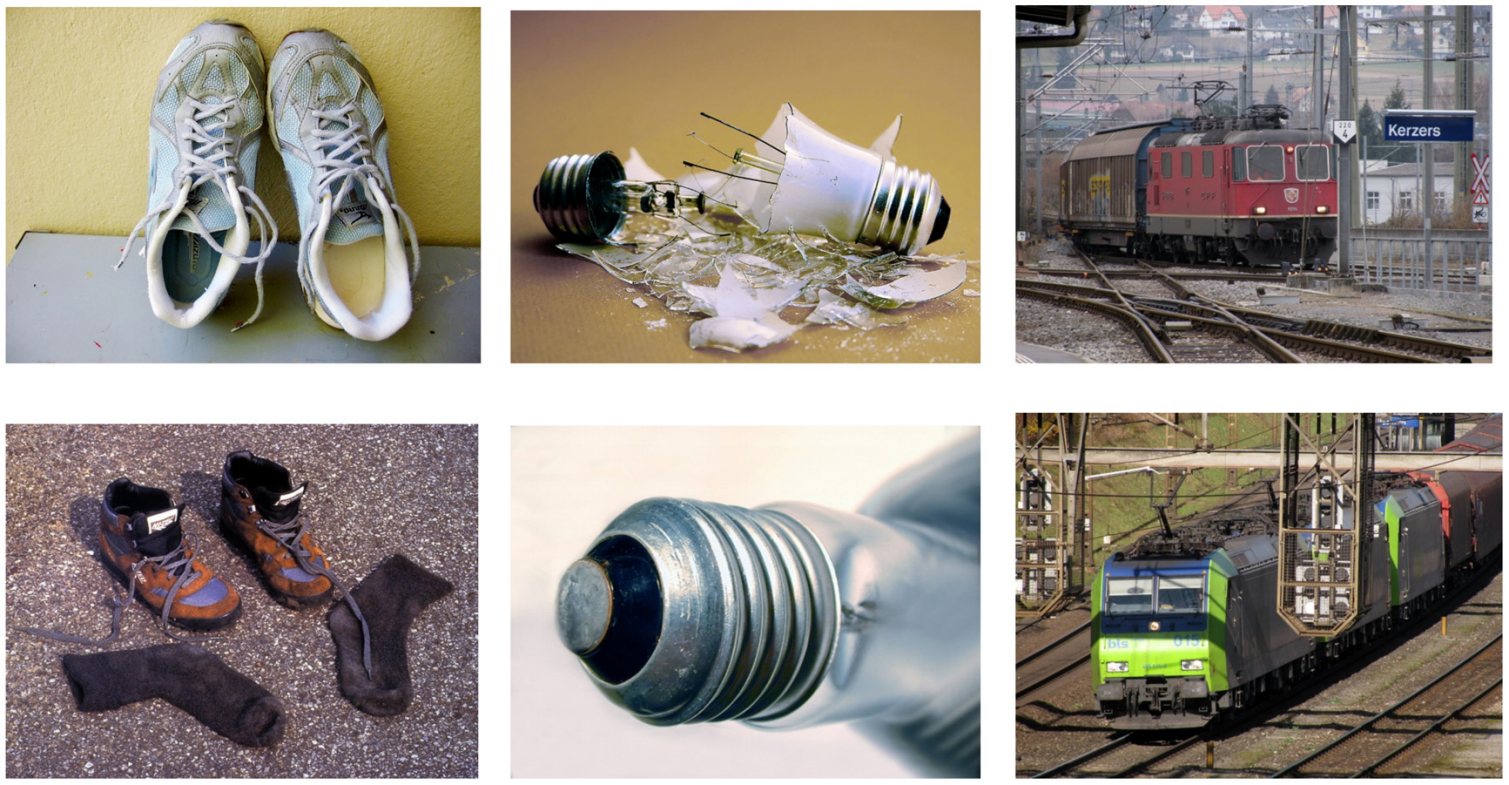
**Illustration of the revised picture sets in experiment 4 showing three representative new target-distracter pairs**.

Fifteen of the 90 image pairs were replaced (see **Figure 5** for examples of replacement pairs).

### Results

#### Hits and False Alarms

Hits and false alarms are given in **Table 4**. A significant main effect was observed for hits [*F*_(2,46)_ = 25.38; *p* < 0.001; $\eta_{p}^{2}$ = 0.53]. TBR hit rate was again significantly higher than TBF and UI hit rates (*p* < 0.001, respectively). Also, TBF hit rate was significantly higher than UI hit rate (*p* < 0.05). No significant effect was found for false alarms [*F*_(2,46)_ = 0.14; *p* = 0.87; $\eta_{p}^{2}$ = 0.01]. False alarm rate did not differ significantly between TBR, TBF, and UI (*p*s > 0.6).

**Table 4:** Experiment 4.

Hits			False Alarms		
	
Remember	Forget	Un-cued	Remember	Forget	Un-cued
0.74^a^	0.58^b^	0.52^c^	0.22^a^	0.21^a^	0.22^a^
(0.02)	(0.03)	(0.04)	(0.02)	(0.02)	(0.02)


*Mean hit and false alarm rates in the ‘remember,’ ‘forget,’ and ‘un-cued’ condition. Standard errors appear in parentheses below means; means in the same row sharing the same superscript letter do not differ significantly from each other, means that do not share subscripts differ at *p* ≤ 0.05.*

#### Discrimination Accuracy and Response Bias

**FIGURE 6:**
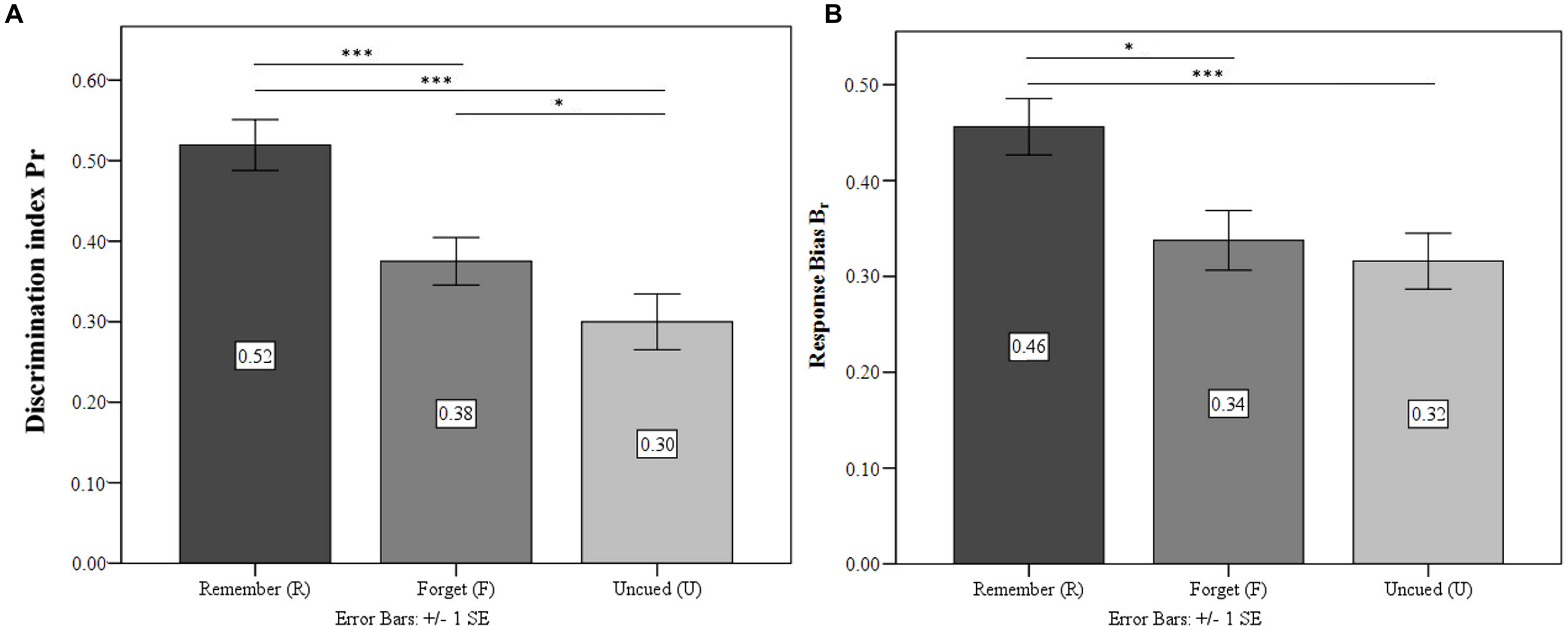
**Experiment 4.** **(A)** Discrimination index for the condition Remember (R), Forget (F), and Un-cued (U). **(B)** Response bias for the three conditions. ^∗^*p* ≤ 0.05, ^∗∗∗^*p* ≤ 0.001.

ANOVA confirmed significant differences in discrimination accuracy *P*_r_ between differently cued stimuli [*F*_(2,46)_ = 27.59; *p* < 0.001; $\eta_{p}^{2}$ = 0.55] and revealed that TBR were recognized significantly more accurately than TBF (*p* < 0.001) and UI (*p* < 0.001). Also, TBF scored higher than UI (*p* < 0.05). As in Experiments 2 and 3, there were also significant differences for recognition bias *B*_r_ [*F*_(1.48,34.11)_ = 9.14; *p* < 0.01; $\eta_{p}^{2}$ = 0.28]. TBR response bias was significantly less conservative than TBF (*p* < 0.05) and UI (*p* < 0.001). TBF and UI did not differ (*p* = 0.49). **Figure 6** illustrates this pattern.

### Discussion Experiment 4

Regarding recognition accuracy, Experiment 4, replicates findings from Experiments 1–3. As in Experiment 1, this effect was mostly carried by hits. The data suggest that difficulty may play a role in whether the consistent accuracy effect is driven by differences in hits or false alarms, possibly reflecting the extent to which participants relied on gist representation. Although list length was longer in Experiment 4 than in Experiment 1, some of the most difficult item pairs were eliminated from Experiment 4, perhaps balancing for effects of list length. In Experiment 4 as in Experiments 2 and 3 the response bias is most lenient for TBR, however, unlike in Experiments 2 and 3, the response bias for UI was as conservative as for TBF. Across all experiments, false alarm rate was always lowest for TBF. No instruction-dependent difference in response bias was found in Experiment 1. Across the experiments, it appears that instruction-dependent differences in recognition accuracy with TBR being remembered more accurately than TBF and crucially TBF more accurately than UI is a robust phenomenon in item-method DF, whereas effects on the recognition bias are more variable. To formally assess similarities and differences between the four experiments and underscore the statistical stability of findings, in a final step across-experiment comparison was conducted for hits and false alarms as well as discrimination accuracy *P*r and recognition bias *B*r.

## Between Studies Comparison

A mixed ANOVA with the between factors Experiment and the within factor Cue (TBR, TBF, UI) and Response Type (hits and false alarms) and two additional separate ANOVAs for discrimination accuracy *P*r and recognition bias *B*r, again with the between factor Experiment and the within factor Cue (TBR, TBF, UI), were calculated for the data from all 122 participants.

### Results

#### Hits and False Alarms

Mean hit and false-alarm rates are detailed in **Table 5**. Overall, a main effect on hits was found [*F*_(2,242)_ = 96.99, *p* < 0.001; $\eta_{p}^{2}$ = 0.45]. TBR hit rate was significantly higher than TBF and UI hit rates (both *p*s < 0.001). Also, TBF hit rate was significantly higher than UI hit rate (*p* < 0.01). Hit rate did not interact with experiment [*F*_(6,236)_ = 0.97, *p* = 0.45; $\eta_{p}^{2}$ = 0.02]. Across experiments, there was a significant main effect on false alarms [*F*_(2,242)_ = 27.58, *p* < 0.001; $\eta_{p}^{2}$ = 0.19]. Overall, false alarm rate was lower for TBF compared to TBR (*p* < 0.05) and UI (*p* < 0.001). TBR false alarm rate was also significantly lower than for UI (*p* < 0.001). False alarm rate differed between experiments [*F*_(6,236)_ = 3.23, *p* < 0.01; $\eta_{p}^{2}$ = 0.08]. False alarm rate was significantly lower in Experiment 1 compared to Experiment 2 (*p* < 0.001) and Experiment 3 (*p* < 0.001). Also, false alarm rate was lower in Experiment 4 compared to Experiment 2 (*p* < 0.05).

**Table 5:** Overall comparisons across the four experiments.

Hits			False Alarms		
	
Remember	Forget	Un-cued	Remember	Forget	Un-cued
0.74^a^	0.60^b^	0.57^c^	0.22^a^	0.20^b^	0.28^c^
(0.01)	(0.02)	(0.02)	(0.01)	(0.01)	(0.01)


*Mean hit and false alarm rates in the ‘remember,’ ‘forget,’ and ‘un-cued’ condition. Standard errors appear in parentheses below means; means in the same row sharing the same superscript letter do not differ significantly from each other, means that do not share subscripts differ at *p* ≤ 0.05.*

#### Discrimination Accuracy and Response Bias

**FIGURE 7:**
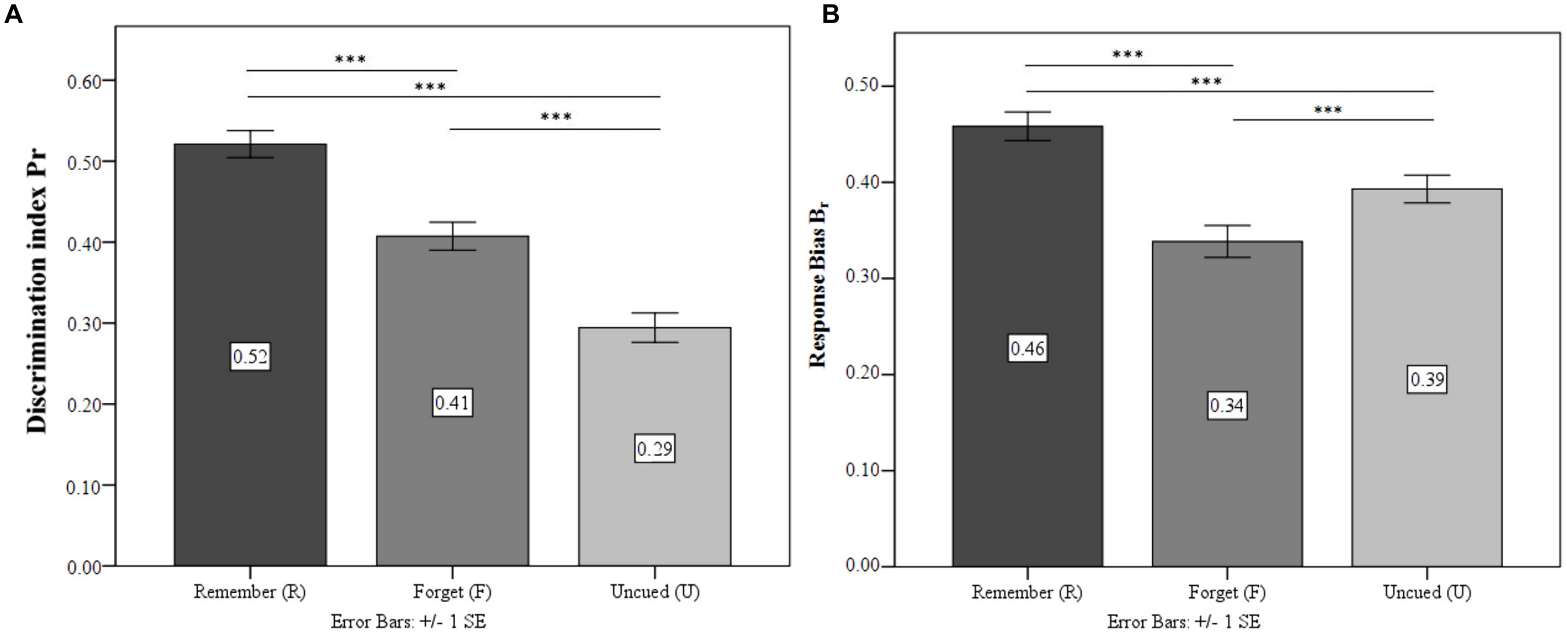
**Overall comparisons across the four experiments.** **(A)** Discrimination index for the condition Remember (R), Forget (F), and Un-cued (U). **(B)** Response bias for the three conditions. ^∗∗∗^*p* ≤ 0.001.

ANOVA confirmed significant differences in discrimination accuracy *P*_r_ between differently cued stimuli [*F*_(2,236)_ = 109.56, *p* < 0.001; $\eta_{p}^{2}$ = 0.48] and revealed that TBR were recognized significantly more accurately than TBF (*p* < 0.001) and UI (*p* < 0.001). Crucially, TBF scored higher than UI (*p* < 0.001). Discrimination accuracy did not differ between experiments [*F*_(6,236)_ = 0.56, *p* = 0.76; $\eta_{p}^{2}$ = 0.01]. Significant differences for recognition bias *B*_r_ were found [*F*_(1.73,203.98)_ = 25.56; *p* < 0.001; $\eta_{p}^{2}$ = 0.18]. TBF response bias was significantly more conservative than TBR (*p* < 0.001) and UI (*p* < 0.001). UI response bias was also significantly more conservative than TBR (*p* = 0.001). Response bias interacted with experiment [*F*_(5.19,203.98)_ = 2.99, *p* < 0.05; $\eta_{p}^{2}$ = 0.07]. Response bias was significantly more conservative in Experiment 1 compared to Experiment 3 (*p* < 0.05). The results for this cross-experiment analysis are shown in **Figure 7**.

## General Discussion

This series of experiments compared recognition memory for items encoded under remember and forget instructions with recognition memory for incidentally encoded items for which no explicit instruction was given. Across four experiments, discrimination accuracy was best for TBR and worst when no specific instruction was given, leaving items to be implicitly encoded. Relative to totally UI, TBF were remembered more accurately, instead of equally well or worse than UI, and this held even when the conditions were fully perceptually matched. Better recognition of TBR than of TBF items is in line with the view that the item-method DF effect might be primarily due to ‘selective rehearsal’ of TBR. Still, selective rehearsal might not be fully able to account for why TBF were recognized better than UI. The forget instruction has been shown to induce inhibition in spatial attention using the IOR paradigm (Taylor, 2005; Taylor and Fawcett, 2011, 2012). However, it does not seem to impair recognition accuracy in the same way as active suppression has been shown to do in the Think-No Think paradigm (e.g., Anderson et al., 2004) or as automatic inhibition does in retrieval-induced forgetting (Anderson et al., 1994). An active memory suppression view of DF is sometimes also adopted in the clinical literature (e.g., Cottencin et al., 2006). Under such a memory suppression account, memory for TBF should be even worse than for incidentally encoded baseline items. Such a pattern might also have occurred, had participants diverted capacities to UI items to distract themselves from TBF as has been shown for item-method DF and the illusionary loudness effect (see Foster and Sahakyan, 2012). The baseline condition involved both mere presentation of UI (Experiments 1 and 2) and additional presentation of perceptually matched symbolic cues (Experiments 3 and 4). Upon testing, UI were consistently recognized less accurately than TBF. Whereas the experiments differed in the extent to which this was due to differences in hits or false alarms, hit rate was never higher for UI than for TBF and false alarm rate was never higher for TBF than for UI. In its traditional version, selective rehearsal can explain more accurate recognition of TBR compared to TBF and UI, but not more accurate recognition of TBF than UI. Conversely, active inhibition effects on recognition memory might predict worse recognition of TBF compared to both TBR and UI. Evidently, in the present experiments active processing of TBF, even with the intention to forget, did not reduce memory to the same extent as no processing instruction at all. Indeed, extending cue-processing time in this paradigm has been shown to improve rather than impair memory for TBF (Bancroft et al., 2013). Thus, whatever active processes occur in item-method DF, these do not necessarily induce successful memory inhibition compared to incidental encoding, although they do result in inhibitory phenomena in other domains (Fawcett and Taylor, 2008; Taylor and Fawcett, 2012; Lee and Hsu, 2013). Accordingly, frontal brain activations previously observed in this design (Hauswald et al., 2011; Nowicka et al., 2011; van Hooff and Ford, 2011; Rizio and Dennis, 2013) may result from either non-inhibitory processes within the frontal lobes, such as conflict monitoring (Silvetti et al., 2014) or attention orienting (Chun and Turk-Browne, 2007) or perhaps from unsuccessful inhibition attempts. The latter view would be consistent with the operation of ironic monitoring (Wegner et al., 1987; Wegner, 1994, 1997; Wenzlaff and Wegner, 2000) as well as findings from cognitive linguistics demonstrating the extra cognitive load of having to process negative statements (Kaup, 2001; Ferguson et al., 2008; Lüdtke et al., 2008). Overall, the forget cue may induce automatic reprocessing of the associated item, causing the present effect. The reality of the findings is underscored by the fact that participants were offered monetary incentive for accurate performance.

Of course, there are ambiguities associated with leaving participants to their own devices in an experiment and presenting material that is not associated with any specific instruction. Behavioral data cannot fully answer the question of what participants actually do when receiving an UI versus an TBF instructions, although incidental encoding situations are quite natural and have been amply used in the literature (e.g., Craik and Lockhart, 1972; Hockley, 2008; Hockley et al., 2015). By some TBF might be considered as even stricter F cues. However, an explicit ignore instruction (as in variants of list-method DF) was never given here, UI were just not commented on. Also, participants could have been confused about the difference between TBF and UI items. We asked participants whether there were problems with the instruction and, at least on the self-report level, there was no indication of confusion. Moreover, data on effects of left prefrontal tDCS stimulation acquired in the context of Experiment 3 (Zwissler et al., 2014) show that for the R and the F conditions cathodal and anodal left prefrontal tDCS stimulation had antagonistic effects on false alarm rate. However, neither anodal nor cathodal tDCS affected the UI condition compared to the sham condition whose data are reported here. This underscores that both F-cued and R-cued induce, albeit qualitatively different, active processes in the prefrontal cortex that are not activated when the perceptual symbol is not associated with a specific memory instruction as in UI. Similarly, EEG data acquired in the context of Experiment 4 indicate qualitative processing differences between all three conditions. In particular, a previously identified frontal positivity, at the time suggested to indicate active inhibition (Hauswald et al., 2011), was larger for F than for UI and R items, the UI positivity being also larger than the frontal R positivity. By contrast, a parietal positivity indexing selective rehearsal was larger for R than for both F and UI items, F being again larger than UI. Both the tDCS and the EEG data are in line with the notion that the F-cue induces active, but regarding recognition memory, non-inhibitory processing which is qualitatively different from the type of processing induced by the R cue. Crucially, F cues result in less effective forgetting than a cue that does not explicitly specify a memory instruction.

It cannot be completely ruled out that participants did not follow the given instructions but instead rehearsed items independently of instruction across an entire set, especially in case of semantically interrelated stimuli (i.e., cars, humans, animals). However, due to the size and the thematic diversity of the image sets, a systematic distortion seems rather unlikely. Finally, in Experiments 3 and 4, we chose to resolve the physical difference between behaviorally relevant cues (R, F) and the irrelevant one (U) by assigning a symbol to each of them. This might raise the question whether a symbol carrying no meaning still qualifies as a non-existent cue. Results do not suggest a major difference between the first and the last experiment. Experimenters can never be quite sure what participants really do, even when they receive an explicit instruction, and the problem might be exacerbated when no instruction is given. On the other hand, free viewing and uninstructed processing is a very natural situation as much of the material that is encountered in everyday life is not associated with explicit instructions and sometimes arguably not even with an intrinsic goal. Therefore having a certain proportion of stimuli that is not associated with an explicit instruction would appear quite natural in many situations. Indeed, the data pattern suggests that across participants there was a systematic response to UI as well as to TBR or TBF. Free viewing has been used in various areas of perception (Junghöfer et al., 2001; Kissler et al., 2007) and memory (Potter and Levy, 1969; Potter, 1976) research. Present data incorporating a free viewing condition indicate that even under fully perceptually matched conditions discrimination accuracy for items not associated with a specific instruction is poorer than for items explicitly instructed to be forgotten and that only these are truly ignored and decay passively.

There were also effects on the response bias: It is notable that these depart from what would be expected under a typical TBR strengthening account. Typically, strengthening items leads to a more conservative response bias (Stretch and Wixted, 1998). Thus, TBR items should have been responded to more conservatively than the other item types, UI showing the most liberal response bias. Yet, TBF were by-and-large responded to most conservatively, which could be indicative of a separate effect of the forget instruction on how participants set their response criterion. Indeed, across experiments false alarm rate was always lowest for TBF. Effects on response bias are generally more apparent with higher false alarm rates and lower hit rates, as in Experiments 2 and 3, where TBF were responded to less readily than TBR and UI. That is, in spite of monetary incentive to the contrary, participants required more mnemonic evidence to make an ‘old’ decision to TBF than to the other item types. The initial forget instruction may induce a subjective underrepresentation of the frequency of forget items on the test list which would have reduced participants’ willingness to endorse these items as old (Strack and Förster, 1995; Hirshman and Henzler, 1998). Perhaps this reflects one aspect of the inhibitory processing found to be induced by the forget instruction in other contexts. Such a bias may be beneficial in legal settings, resulting in a reduced tendency to misidentify look-alikes of an exonerated former suspect from a line-up. Unfortunately, for a mere bystander (UI in our context), misidentification tendencies might be higher at least under some circumstances. Further research will specify how different memory instructions interact with other experimental parameters in item-method DF.

Even where it occurs, a conservative response bias apparently cannot compensate for the initial alerting process. As a consequence, both TBR and TBF are remembered more accurately than UI. Several findings (e.g., Lee et al., 2007; Fawcett and Taylor, 2008, 2010) suggest that TBF are not instantaneously toned down during learning. Rather, even TBF benefit from longer post-cue intervals. Presumably, when a stimulus is presented, it is being held online to begin with. After onset of a ‘meaningful’ cue (i.e., R and F), both these stimulus types receive special attention. Only for UI, it seems that processing ceases after stimulus off-set. This happens even when UI are followed by a perceptually equivalent symbolic cue to which no cognitive significance is assigned. The effect is seen in each individual experiment and underscored by the cross-experiment analysis, where it is seen for both hit rate and discrimination accuracy with no cross-experiment interaction. Still, visually the above experiments differ in the extent to which this effect is carried by hits versus false alarms. Future research will further specify the dynamics of the present phenomenon, however, tentatively, list length and overall target discrimination levels could be important factors.

The present results may appear surprising in view of experimental evidence of successful representational inhibition of target items compared to baseline in the Think-No Think paradigm (Anderson and Green, 2001; Anderson et al., 2004). In the Think-No Think paradigm and in item-method DF as in cognitive control in general, prefrontal structures have been shown to be involved (Mitchell et al., 2007; Wylie et al., 2008; Giuliano and Wicha, 2010). In DF, prefrontal cerebral activity during cue presentation differentiates intentionally forgotten from incidentally forgotten items (Wylie et al., 2008). Further research will resolve whether the F-instruction’s paradoxical effect is solely due to a short-lived alerting elicited by the F cue. Incorporating un-cued baseline stimuli in neuroscientific studies of DF will aid interpretation of previously observed effects.

Of note, the present studies all used pictorial material and did not test free recall. An important extension of this work will concern the question whether similar results can be found with verbal material and in free recall. So far, data suggest that in item-method DF, pictorial and verbal materials behave in similar ways (Hourihan et al., 2009; Quinlan et al., 2010), but firm conclusions await further empirical tests. Also, the use of thematically matched pairs may have been problematic. As in some previous research (Zwissler et al., 2011, 2012), this approach had been used to facilitate scoring of hits and false alarms per item category. However, participants may have noticed that the material was organized in pairs and this may have biased their responses in unforseeable ways. The most obvious possibility is that participants on presentation of the second picture from such a thematic pair realized that they had gotten the first one wrong because they had made a gist-based decision. While this may have helped them on the second decision, they could not undo the first response and therefore the procedure enhanced noise in the data. Most likely, such noise would be distributed equally across all conditions. Still, there is the possibility that such effects interacted with instruction in hitherto unknown ways.

For the current methods and materials, the current study raises the possibility that item-method DF could involve ironic processes. Initially, two operations may be required: one to remember TBR, which is a common task for students; the second is to forget TBF, which is comparably unusual. As Wegner (1994) suggests, under mental load resources are drawn from the operating process and an ironic monitoring process takes over interfering with thought control, or presently, with successful forgetting.

The present research demonstrates that item-method DF occurs only in comparison to a ‘remember’ instruction and not compared to giving no instruction at all. Thus, regarding recognition accuracy, the F-cue induces active, but not inhibitory processing. These results are in line with other findings demonstrating that humans have trouble processing negative information and have practical implications for educational and legal settings.

## Conflict of Interest Statement

The authors declare that the research was conducted in the absence of any commercial or financial relationships that could be construed as a potential conflict of interest.

## Footnotes

1 In old/new recognition memory experiments, hits and false alarms need to be considered simultaneously to yield measures of memory accuracy on the one hand and response bias on the other as participants’ recognition data will be determined both by the actual memory strength for an item and their readiness to respond given a certain amount of mnemonic information. Several such models have been developed (see Snodgrass and Corwin, 1988). Here, we chose the two-high threshold model. However, calculation of the *d*′ and *C* measures reveals equivalent results. For a discussion of the relative merits of different models of recognition memory, see e.g., (Broder and Schutz, 2009).

2 Data from this experiment have been partly reported by Zwissler et al. (2014) in the context of a study on tDCS modulations of memory accuracy. The presently reported data are from the sham condition in Zwissler et al. (2014).

## References

[B1] Anderson M. C. Bjork R. A. Bjork E. L. (1994). Remembering can cause forgetting: retrieval dynamics in long-term memory. *J. Exp. Psychol. Learn. Mem. Cogn.* 20 1063–1087. 10.1037/0278-7393.20.5.1063 7931095

[B2] Anderson M. C. Green C. (2001). Suppressing unwanted memories by executive control. *Nature* 410 366–369. 10.1038/35066572 11268212

[B3] Anderson M. C. Hanslmayr S. (2014). Neural mechanisms of motivated forgetting. *Trends Cogn. Sci.* 18 279–292. 10.1016/j.tics.2014.03.002 24747000 PMC4045208

[B4] Anderson M. C. Ochsner K. N. Kuhl B. Cooper J. Robertson E. Gabrieli S. W. (2004). Neural systems underlying the suppression of unwanted memories. *Science* 303 232–235. 10.1126/science.1089504 14716015

[B5] Bancroft T. D. Hockley W. E. Farquhar R. (2013). The longer we have to forget the more we remember: the ironic effect of postcue duration in item-based directed forgetting. *J. Exp. Psychol. Learn. Mem. Cogn.* 39 691–699. 10.1037/a0029523 22845067

[B6] Basden B. H. Basden D. R. (1996). Directed forgetting: further comparisons of the item and list methods. *Memory* 4 633–653. 10.1080/741941000 8934458

[B7] Basden B. H. Basden D. R. (1998). “Directed forgetting: a contrast of methods and interpretations,” in *Intentional Forgetting: Interdisciplinary Approaches*, eds Golding J. M. MacLeod C. M. (Mahwah, NJ: Lawrence Erlbaum Associates).

[B8] Baumann M. Zwissler B. Schalinski I. Ruf-Leuschner M. Schauer M. Kissler J. (2013). Directed forgetting in post-traumatic-stress-disorder: a study of refugee immigrants in Germany. *Front. Behav. Neurosci.* 7:94 10.3389/fnbeh.2013.00094 PMC3736047 23966914

[B9] Botvinick M. M. Braver T. S. Barch D. M. Carter C. S. Cohen J. D. (2001). Conflict monitoring and cognitive control. *Psychol. Rev.* 108 624–652. 10.1037/0033-295X.108.3.624 11488380

[B10] Brickenkamp R. (1994). *Test d2 Aufmerksamkeits-Belastungs-Test [Attention Test]*. Göttingen: Hogrefe.

[B11] Broder A. Schutz J. (2009). Recognition ROCs are curvilinear-or are they? On premature arguments against the two-high-threshold model of recognition. *J. Exp. Psychol. Learn. Mem. Cogn.* 35 587–606. 10.1037/a0015279 19379038

[B12] Chun M. M. Turk-Browne N. B. (2007). Interactions between attention and memory. *Curr. Opin. Neurobiol.* 17 177–184. 10.1016/j.conb.2007.03.005 17379501

[B13] Cottencin O. Vaiva G. Huron C. Devos P. Ducrocq F. Jouvent R. (2006). Directed forgetting in PTSD: a comparative study versus normal controls. *J. Psychiatr. Res.* 40 70–80. 10.1016/j.jpsychires.2005.04.001 15907941

[B14] Craik F. I. Lockhart R. S. (1972). Levels of processing: a framework for memory research. *J. Verbal Learn. Verbal Behav.* 11 671–684. 10.1016/S0022-5371(72)80001-X

[B15] Fawcett J. M. Taylor T. L. (2008). Forgetting is effortful: evidence from reaction time probes in an item-method directed forgetting task. *Mem. Cogn.* 36 1168–1181. 10.3758/MC.36.6.1168 18927035

[B16] Fawcett J. M. Taylor T. L. (2010). Directed forgetting shares mechanisms with attentional withdrawal but not with stop-signal inhibition. *Mem. Cogn.* 38 797–808. 10.3758/MC.38.6.797 20852242

[B17] Ferguson H. J. Sanford A. J. Leuthold H. (2008). Eye-movements and ERPs reveal the time course of processing negation and remitting counterfactual worlds. *Brain Res.* 1236 113–125. 10.1016/j.brainres.2008.07.099 18722356

[B18] Foster N. L. Sahakyan L. (2012). Metacognition influences item-method directed forgetting. *J. Exp. Psychol. Learn. Mem. Cogn.* 38 1309–1324. 10.1037/a0027868 22468801

[B19] Giuliano R. J. Wicha N. Y. Y. (2010). Why the white bear is still there: electrophysiological evidence for ironic semantic activation during thought suppression. *Brain Res.* 1316 62–74. 10.1016/j.brainres.2009.12.041 20044982 PMC2822038

[B20] Golding J. M. MacLeod C. M. (1998). *Intentional Forgetting: Interdisciplinary Approaches*. Mahwah, NJ: Lawrence Erlbaum Associates Publishers.

[B21] Hauswald A. Kissler J. (2008). Directed forgetting of complex pictures in an item method paradigm. *Memory* 16 797–809. 10.1080/09658210802169087 18608977

[B22] Hauswald A. Schulz H. Iordanov T. Kissler J. (2011). ERP dynamics underlying successful directed forgetting of neutral but not negative pictures. *Soc. Cogn. Affect. Neurosci.* 6 450–459. 10.1093/scan/nsq061 20601423 PMC3150854

[B23] Hirshman E. (1995). Decision processes in recognition memory: criterion shifts and the list-strength paradigm. *J. Exp. Psychol. Learn. Mem. Cogn.* 21 302–313. 7738502 10.1037//0278-7393.21.2.302

[B24] Hirshman E. Henzler A. (1998). The role of decision processes in conscious recollection. *Psychol. Sci.* 9 61–65. 10.1111/1467-9280.00011

[B25] Hockley W. E. (2008). The effects of environmental context on recognition memory and claims of remembering. *J. Exp. Psychol. Learn. Mem. Cogn.* 34 1412–1429. 10.1037/a0013016 18980405

[B26] Hockley W. E. Ahmad F. N. Nicholson R. (2015). Intentional and incidental encoding of item and associative information in the directed forgetting procedure. *Mem. Cogn.* 1–9. 10.3758/s13421-015-0557-8 26407851

[B27] Hourihan K. L. Ozubko J. D. MacLeod C. M. (2009). Directed forgetting of visual symbols: evidence for non-verbal selective rehearsal. *Mem. Cogn.* 37 1059–1068. 10.3758/MC.37.8.1059 19933451

[B28] Hourihan K. L. Taylor T. L. (2006). Cease remembering: control processes in directed forgetting. *J. Exp. Psychol. Hum. Percept. Perform.* 32 1354–1365. 17154777 10.1037/0096-1523.32.6.1354

[B29] Junghöfer M. Bradley M. M. Elbert T. R. Lang P. J. (2001). Fleeting images: a new look at early emotion discrimination. *Psychophysiology* 38 175–178. 10.1111/1469-8986.3820175 11347862

[B30] Kaup B. (2001). Negation and its impact on the accessibility of text information. *Mem. Cogn.* 29 960–967. 10.3758/BF03195758 11820755

[B31] Kissler J. Herbert C. Peyk P. Junghofer M. (2007). Buzzwords: early cortical responses to emotional words during reading. *Psychol. Sci.* 18 475–480. 10.1111/j.1467-9280.2007.01924.x 17576257

[B32] Lee Y.-S. Hsu Y. C. (2013). How do we forget negative events? The role of attentional, cognitive, and metacognitive control. *Cogn. Emot.* 27 401–415. 10.1080/02699931.2012.713326 22894763

[B33] Lee Y.-S. Lee H.-M. Fawcett J. M. (2013). Intentional forgetting reduces color-naming interference: evidence from item-method directed forgetting. *J. Exp. Psychol. Learn. Mem. Cogn.* 39 220–236. 10.1037/a0028905 22732028

[B34] Lee Y.-S. Lee H.-M. Tsai S.-H. (2007). Effects of post-cue interval on intentional forgetting. *Br. J. Psychol.* 98 257–272. 10.1348/000712606X120410 17456272

[B35] Lehman E. B. McKinley-Pace M. Leonard A. M. Thompson D. Johns K. (2001). Item-cued directed forgetting of related words and pictures in children and adults: selective rehearsal versus cognitive inhibition. *J. Gen. Psychol.* 128 81–97. 10.1080/00221300109598900 11277450

[B36] Levy B. J. Anderson M. C. (2009). “The control of mnemonic awareness,” in *Encyclopedia of Consciousness*, ed. Banks W. P. (San Diego, CA: Elsevier).

[B37] Lüdtke J. Friedrich C. K. De Filippis M. Kaup B. (2008). Event-related potential correlates of negation in a sentence-picture verification paradigm. *J. Cogn. Neurosci.* 20 1355–1370. 10.1162/jocn.2008.20093 18303972

[B38] MacLeod C. M. (1999). The item and list methods of directed forgetting: test differences and the role of demand characteristics. *Psychon. Bull. Rev.* 6 123–129. 10.3758/BF03210819 12199306

[B39] Mitchell J. P. Heatherton T. F. Kelley W. M. Wyland C. L. Wegner D. M. Macrae C. N. (2007). Separating sustained from transient aspects of cognitive control during thought suppression. *Psychol. Sci.* 18 292–297. 10.1111/j.1467-9280.2007.01891.x 17470250

[B40] Nowicka A. Marchewka A. Jednoróg K. Tacikowski P. Brechmann A. (2011). Forgetting of emotional information is hard: an fMRI study of directed forgetting. *Cereb. Cortex* 21 539–549. 10.1093/cercor/bhq117 20584747

[B41] Paz-Caballero M. D. Menor J. Jimenez J. M. (2004). Predictive validity of event-related potentials (ERPs) in relation to the directed forgetting effects. *Clin. Neurophysiol.* 115 369–377. 10.1016/j.clinph.2003.09.011 14744579

[B42] Potter M. C. (1976). Short-term conceptual memory for pictures. *J. Exp. Psychol. Hum. Learn.* 2 509–522. 10.1037/0278-7393.2.5.509 1003124

[B43] Potter M. C. Levy E. I. (1969). Recognition memory for a rapid sequence of pictures. *J. Exp. Psychol.* 81 10–15. 10.1037/h0027470 5812164

[B44] Quinlan C. K. Taylor T. L. Fawcett J. M. (2010). Directed forgetting: comparing pictures and words. *Can. J. Exp. Psychol.* 64 41–46. 10.1037/a0016569 20384417

[B45] Rhodes M. G. Castel A. D. (2009). Metacognitive illusions for auditory information: effects on monitoring, and control. *Psychon. Bull. Rev.* 16 550–554. 10.3758/PBR.16.3.550 19451383

[B46] Rizio A. A. Dennis N. A. (2013). The neural correlates of cognitive control: successful remembering and intentional forgetting. *J. Cogn. Neurosci.* 25 297–312. 10.1162/jocn_a_00310 23066730

[B47] Silvetti M. Alexander W. Verguts T. Brown J. W. (2014). From conflict management to reward-based decision making: actors and critics in primate medial frontal cortex. *Neurosci. Biobehav. Rev.* 46 44–57. 10.1016/j.neubiorev.2013.11.003 24239852

[B48] Snodgrass J. G. Corwin J. (1988). Pragmatics of measuring recognition memory: applications to dementia and amnesia. *J. Exp. Psychol. Gen.* 117 34–50. 10.1037/0096-3445.117.1.34 2966230

[B49] Strack F. Förster J. (1995). Reporting recollective experiences: direct access to memory systems? *Psychol. Sci.* 6 352–358. 10.1111/j.1467-9280.1995.tb00525.x

[B50] Stretch V. Wixted J. T. (1998). Decision rules for recognition memory confidence judgments. *J. Exp. Psychol. Learn. Mem. Cogn.* 24 1397–1410. 9835060 10.1037//0278-7393.24.6.1397

[B51] Taylor T. L. (2005). Inhibition of return following instructions to remember and forget. *Q. J. Exp. Psychol.* 58 613–629. 10.1080/02724980443000115 16104098

[B52] Taylor T. L. Fawcett J. M. (2011). Larger IOR effects following forget than following remember instructions depend on exogenous attentional withdrawal and target localization. *Atten. Percept. Psychophys.* 73 1790–1814. 10.3758/s13414-011-0146-2 21618066

[B53] Taylor T. L. Fawcett J. M. (2012). Does an instruction to forget enhance memory for other presented items? *Conscious. Cogn.* 21 1186–1197. 10.1016/j.concog.2012.05.002 22687390

[B54] Taylor T. L. Hamm J. P. (2015). Selection for encoding: no evidence of greater attentional capture following forget than remember instructions. *Atten. Percept. Psychophys.* 1–19. 10.3758/s13414-015-0984-4 26404529

[B55] Thompson K. M. Hamm J. P. Taylor T. L. (2014). Effects of memory instruction on attention and information processing: further investigation of inhibition of return in item-method directed forgetting. *Atten. Percept. Psychophys.* 76 322–334. 10.3758/s13414-013-0584-0 24282134

[B56] Thompson K. M. Taylor T. L. (2015). Memory instruction interacts with both visual and motoric inhibition of return. *Atten. Percept. Psychophys.* 77 804–818. 10.3758/s13414-014-0820-2 25592783

[B57] van Hooff J. C. Ford R. M. (2011). Remember to forget: ERP evidence for inhibition in an item-method directed forgetting paradigm. *Brain Res.* 1392 80–92. 10.1016/j.brainres.2011.04.004 21514571

[B58] Wegner D. M. (1994). Ironic processes of mental control. *Psychol. Rev.* 101 34–52. 10.1037/0033-295X.101.1.34 8121959

[B59] Wegner D. M. (1997). When the antidote is the poison: ironic mental control processes. *Psychol. Sci.* 8 148–150. 10.1111/j.1467-9280.1997.tb00399.x

[B60] Wegner D. M. Schneider D. J. Carter S. R. White T. L. (1987). Paradoxical effects of thought suppression. *J. Pers. Soc. Psychol.* 53 5–13. 10.1037/0022-3514.53.1.5 3612492

[B61] Weiner B. (1968). Motivated forgetting and the study of repression. *J. Pers.* 36 213–234. 10.1111/j.1467-6494.1968.tb01470.x 5660729

[B62] Wenzlaff R. M. Wegner D. M. (2000). Thought suppression. *Annu. Rev. Psychol.* 51 59–91. 10.1146/annurev.psych.51.1.59 10751965

[B63] Wylie G. R. Foxe J. J. Taylor T. L. (2008). Forgetting as an active process: an FMRI investigation of item-method-directed forgetting. *Cereb. Cortex* 18 670–682. 10.1093/cercor/bhm101 17617657

[B64] Zacks R. T. Hasher L. (1994). “Directed ignoring: inhibitory regulation of working memory,” in *Inhibitory Mechanisms in Attention, Memory, and Language*, eds Dagenbach D. Carr T. H. (New York, NY: Academic Press), 241–264.

[B65] Zwissler B. Hauswald A. Koessler S. Ertl V. Pfeiffer A. Wöhrmann C. (2012). Memory control in post-traumatic stress disorder: evidence from item method directed forgetting in civil war victims in Northern Uganda. *Psychol. Med.* 42 1283–1291. 10.1017/S0033291711002273 22011378

[B66] Zwissler B. Koessler S. Engler H. Schedlowski M. Kissler J. (2011). Acute psycho-social stress does not disrupt item-method directed forgetting, emotional stimulus content does. *Neurobiol. Learn. Mem.* 95 346–354. 10.1016/j.nlm.2011.01.007 21295148

[B67] Zwissler B. Sperber C. Aigeldinger S. Schindler S. Kissler J. Plewnia C. (2014). Shaping memory accuracy by left prefrontal transcranial direct current stimulation. *J. Neurosci.* 34 4022–4026. 10.1523/JNEUROSCI.5407-13.2014 24623779 PMC3951698

